# Imaging the Visual Pathway in Neuromyelitis Optica

**DOI:** 10.1155/2011/869814

**Published:** 2011-03-16

**Authors:** Caspar F. Pfueller, Friedemann Paul

**Affiliations:** NeuroCure Clinical Research Center, Charité Universitätsmedizin Berlin, 10117 Berlin, Germany

## Abstract

The focus of this paper is to summarize the current knowledge on visual pathway damage in neuromyelitis optica (NMO) assessed by magnetic resonance imaging (MRI) and optical coherence tomography (OCT).

## 1. Introduction

Neuromyelitis optica (NMO, Devic's syndrome) is a rare inflammatory autoimmune central nervous system (CNS) disorder clinically characterised by mostly severe attacks to the optic nerves and the spinal cord [1]. For a long time, NMO was regarded as variant of multiple sclerosis (MS); however, recent increasing evidence points to a distinct pathogenesis. A milestone was the detection of a highly specific biomarker for NMO, the so-called NMO-IgG, the target antigen of which was shown to be the most abundant CNS water channel aquaporin-4 (AQP4) [2, 3]. Various assays for the detection of antibodies to AQP4 have since been developed which has facilitated the clinically relevant distinction of NMO from MS [4, 5]. Antibodies to AQP4 are detectable in 60 to 90% of NMO patients with a specificity of 91–100%. In contrast to previous beliefs, NMO is today regarded as a relapsing disease in 80–90% of patients. With the detection of AQP4 antibodies, the clinical spectrum of NMO has broadened, and currently also AQP4-positive longitudinally extensive transverse myelitis (LETM) and AQP4-positive recurrent optic neuritis are regarded as part of NMO spectrum disorders (NMOSDs) [1, 6]. 

Studies in MS have shown that neuroinflammation and neurodegeneration can lead to an affection of different parts of the visual pathway, including intraretinal inflammation, retinal neurodegeneration, retinal nerve axonal degeneration as well as pathological changes in the optic tract, the lateral geniculate nucleus, the optic radiation, and the visual cortex [7–11]. 

The aim of this paper is to give an overview on the recent research results on a similar or different involvement of the visual pathway in NMO pathology as assessed by magnetic resonance imaging (MRI) and optical coherence tomography (OCT).

## 2. Visual Dysfunction in NMO

In comparison to optic neuritis (ON) in MS, attacks to the optic nerve in NMO occur more often bilaterally and show a poor and incomplete remission of visual functions despite anti-inflammatory and immunosuppressive treatment. At a mean disease duration of 7.7 years after disease onset, more than half of patients with relapsing NMO are functionally blind in at least one eye [12].

## 3. Imaging

### 3.1. Magnetic Resonance Imaging

#### 3.1.1. Spinal Cord

In case of myelitis, spinal cord MRI may reveal a characteristic feature: centrally located longitudinal lesions expanding over 3 or more vertebral segments which may be associated with cord swelling and enhancement with intravenous gadolinium administration. These findings have—in combination with a nondiagnostic brain MRI at disease onset—a specificity of 90% for NMO [13].

#### 3.1.2. Brain

From a clinical standpoint, abnormal brain MRI was until recently considered to argue against an NMO diagnosis. However, several publications in the past few years have highlighted that in NMO various brain lesions may be present at onset or during the course of the disease. A high percentage of NMO patients develop nonspecific brain lesions after diagnosis (60% in the series by Pittock et al. [14], 85% in a Chinese series by Li et al. [15], and in 10% of 60 patients brain lesions met the diagnostic criteria for MS [14]. Another 10% have brain lesions in periependymal regions (e.g., hypothalamus, periaqueductal brainstem) which are rich in AQP4 [15–19]. In contrast to MS, extensive and diffuse widespread white matter abnormalities have been described by various groups. Recently, also large edematous callosal lesions were reported in 4 of 22 NMO patients [20].

Additional MR techniques which are not routinely implemented in clinical practice are magnetization transfer imaging (MTI), diffusion tensor imaging (DTI), and MR spectroscopy (MRS). MTI is a technique that analyses the energy transfer from matrix bound water to free water hydrogen nuclei and thereby allows to evaluate the integrity of an anatomical structure. DTI measures the direction-dependent diffusibility restriction of water, and the resulting data can be computed to characterise fibrous structures, like the optic radiation. MRS is different from other imaging techniques, as it does not provide structural information, but information about ongoing metabolic processes by a concentration measurement of metabolites representative for energy metabolism, integrity of cell membranes, axonal or neuronal integrity and others. These methods have revealed damage to the normal-appearing gray matter but no [21–24] or only minimal abnormalities in the normal-appearing white matter [25]. In contrast, a recent DTI study by Yu and colleagues [26] reported an increased diffusivity of the corticospinal tract and the optic radiations but not of the corpus callosum and the cingulum in NMO patients compared to controls, and the authors concluded that the abnormal diffusion was restricted to the regions with connections to the optic nerves and the spinal cord, thus arguing for axonal degeneration secondary to lesions in the optic nerve and the spinal cord.

#### 3.1.3. Visual Pathway: Optic Nerve and Optic Radiation

MR Imaging studies aimed to visualize optic nerve damage in NMO are sparse. Li et al. reported optic nerve sheath thickening in 16 of 33 patients, all of whom had symptoms of recurrent ON [15]. Wang and colleagues found optic nerve hyperintensities in 6 of 10 patients with available optic nerve MRI [19]. Enhancement of the optic nerves with intravenous gadolinium administration was detected in 4 patients in whom MRI was performed within 6 weeks of onset of acute ON. Similar observations have been made in a comparable study [27]. However, as similar findings have been described in MS optic neuritis [28], it is doubtful whether the aforementioned findings are specific features of NMO or rather indicate optic nerve inflammation irrespective of the underlying condition. Lin et al. reported the possibility to discriminate NMO from MS based on DTI data. However, no tract-specific analysis was performed here [29]. The only MRI study to date that assessed the retrogeniculate part of the visual pathway in NMO by DTI reported an increased diffusivity of the optic radiation [26]. To our knowledge, there is no bigger study on MT imaging of the visual pathway in NMO patients.

### 3.2. Optical Coherence Tomography

OCT is a noninvasive and reproducible technique to study unmyelinated retinal axons with a high spatial resolution *in vivo* and to quantify the thickness of the peripapillary retinal nerve fiber layer (RNFL), fovea, and macula [30]. In MS patients, OCT has been consistently shown to detect thinning of the RNFL which is most probably due to a diffuse damage of retinal axons that occurs at least in part independent of a previous or present attack of ON [31–33]. Against this background, OCT might prove as a valuable tool for the detection and monitoring of axonal damage in MS and other inflammatory CNS conditions such as NMO. Several groups have investigated anterior visual pathway damage in NMO by OCT in comparison to healthy controls or MS patients. The first published study on OCT in NMO by de Seze and colleagues [34] reported dramatically reduced average RNFL thickness in NMO patients as compared to healthy controls (77.9 *μ*m versus 102.3 *μ*m) and a good correlation between OCT results and both visual acuity and visual evoked potential latencies. Interestingly, among patients at high risk for NMO (recurrent ON of LETM), only those with recurrent ON had a similarly severe reduction of average RNFL thickness (74.2 *μ*m) in contrast to those with recurrent myelitis who did not differ from controls with respect to average RNFL thickness (101.8 *μ*m versus 102.3 *μ*m). Another recent OCT study by Naismith and colleagues compared RNFL measurements in NMO patients to MS [35]. In accordance with the clinical experience of a more severe loss of visual function in NMO compared to MS following ON, RNFL thickness was significantly thinner in NMO patients than in MS ones after ON suggesting a more profound axon loss in the optic nerve in NMO. Similar results were obtained by Ratchford et al. [36] who estimated a first episode of ON to cause 24 *μ*m more loss of RNFL thickness in NMO than in relapsing-remitting MS. Moreover, also macular volume was significantly reduced in NMO ON eyes both versus MS and healthy controls. Interestingly, eyes in the subgroup of patients with LETM and unaffected NMO eyes were not different from controls. The difference in RNFL thickness between ON and non-ON eyes was much greater in NMO patients than in MS ones (34.3 *μ*m versus 9.6 *μ*m). This may suggest that retinal axonal damage in NMO is predominantly linked to attacks of ON while in MS thinning of the RNFL has been reported—albeit to a lesser extent—also in non-ON eyes [32, 33]. Other hypothetical explanations may be a more frequent occurrence of subclinical optic neuritis in MS than in NMO, or MS lesions in the chiasm or optic tracts causing bilateral RNFL involvement [35]. 

It is worth to critically discuss the significance of OCT for monitoring axonal damage. On one hand the validity of OCT measurements in the past was limited by device-specific measurement variations of the time-domain tomographs in the range of a suspected effect, for example, for RNFLT changes over time. It is expected that the novel spectral-domain OCT devices provide an improved spatial resolution and a better retest-reliability in the future and thereby help to give a more precise description of damage to the visual pathway and its underlying pathogenetic correlate [37, 38]. 

On the other hand, there is increasing evidence that OCT measurements do not reflect axonal damage exclusively, but could also be affected by intraretinal inflammation or retinal neuronal degeneration, as described in a recent neuropathological study on postmortem analysis on postmortem MS brain tissue by Green et al. [7].

### 3.3. Is There an Interplay of Damage to the Anterior and the Posterior Visual Pathway?

Imaging the entire visual pathway in inflammatory CNS conditions with clinical optic nerve involvement may provide the opportunity to study the relationship between damage to the anterior (optic nerves, chiasm, optic tracts) and attrition to the posterior visual pathway (lateral geniculate nucleus (LGN), optic radiations, visual cortex in the occipital lobe). It is conceivable that damage to one part of the visual pathway may cause—via antero- or retrograde transsynaptic degeneration—an alteration in the other part of the visual pathway. In the past decades, transsynaptic degeneration in the visual system has been shown in various animal models [39–43]. A recent OCT study showed significant RNFL thinning in patients with retrogeniculate lesions (congenital or acquired occipital lobe damage) [44]. In MS, a histopathological study revealed neuronal loss in the LGN [9]. Several other groups have since addressed the question of transsynaptic degeneration by different imaging techniques in MS patients *in vivo*. Sepulcre and colleagues demonstrated that atrophy of the LGN was related to the presence of lesions specifically in the optic radiations but not in the rest of the brain [10]. Ciccarelli et al. examined patients one year after an episode of ON and found reduced connectivity in the optic radiations, suggesting both transsynaptic effects and axonal loss in the optic radiations related to LGN neuronal loss [45]. Audoin et al. reported a decreased visual cortex magnetization transfer ratio in patients with optic neuritis [11]. Dasenbrock et al., in a combined DTI-OCT study in patients with MS and healthy controls, reported a significant association of optic tract diffusion abnormalities with RNFL thinning [46]. In a large cross-sectional study comprising 86 relapsing-remitting MS patients, we investigated the association of RNFL changes with brain atrophy (“brain parenchymal fraction”, BPF) and n-acetyl-aspartate (NAA) concentrations as a marker of neuroaxonal integrity in the visual cortex and the normal-appearing white matter (NAWM) (Pfueller et al., in revision). We found a significant correlation of RNFL thickness with visual cortex NAA, and patients with a previous history of ON on one or both eyes had significantly lower visual cortex NAA values than patients without history of optic neuritis. In contrast, NAWM NAA did not correlate with RNFL thickness, and there was no difference in NAWM NAA between patients with and without ON. BPF also correlated with RNFL thickness, and in a multivariate analysis, both BPF and visual cortex NAA were independently associated with RNFL thickness. Our data suggest that attacks to the optic nerve in MS have a detrimental impact on parts of the visual pathway as remote as the visual cortex, further supporting the assumption of transsynaptic degeneration in the visual pathway.

In NMO, no studies have to date assessed the entire visual pathway by a combination of various imaging techniques (e.g., OCT and optic nerve MRI for the anterior visual pathway, DTI or MRS for the retrogeniculate visual pathway). It is, however, conceivable, that damage to one part of the visual pathway has an impact on the other part also in NMO, as transsynaptic degeneration has been shown in different neurological conditions as delineated above and also in ophthalmologic diseases such as glaucoma [47], (reviewed in [48]). The study by Yu and colleagues showing increased diffusivity in the optic radiations of NMO patients supports this notion [26]. Studying the entire visual pathway by a combination of OCT and different MRI techniques may provide additional insights into the interplay between damage to the anterior and the retrogeniculate part of the visual pathway, as these measurements are far less likely to be confounded by focal inflammatory and demyelinating lesions in the brain as is the case in MS. The absence of these lesions should also facilitate the volumetric analysis of different parts of the visual pathway to address the hypothesis of transsynaptical degeneration and also to assess the dynamics of degenerative processes triggered by single inflammatory events, such as acute optic neuritis. To our knowledge, this approach has not been addressed systematically in a larger cohort of NMO patients. 

To summarize, the repertoire of available imaging techniques (including DTI, MTI, and MR spectroscopy) has not been used yet to its full potential; especially studies focusing on the visual pathway and its anatomical correlates are still missing. Addressing these points could help to identify significant differences between NMO and MS and give further insight into NMO-specific damage processes as well as into transsynaptical damage processes independent of their underlying condition. Of special interest will be the question, whether neurodegeneration in NMO can only occur in the context of acute or previous neuroinflammation or whether there is evidence for diffuse, chronic axonal, and neuronal degeneration independent of acute neuroinflammation, as was shown for MS.
